# A comparison of traditional plant knowledge between students and herders in northern Kenya

**DOI:** 10.1186/s13002-016-0121-z

**Published:** 2016-10-13

**Authors:** Brett L. Bruyere, Jonathan Trimarco, Saruni Lemungesi

**Affiliations:** Human Dimensions of Natural Resources Department, Colorado State University, 1480 Campus Delivery, Fort Collins, CO 80523 USA

**Keywords:** Samburu, Kenya, Local knowledge, Traditional ecological knowledge, Moran, Pastoralism, Plants

## Abstract

**Background:**

The Samburu region of northern Kenya is undergoing significant change, driven by factors including greater value on formal education, improvements in infrastructure and development, a shift from community to private ownership of land, increased sedentary lifestyles and global climate change. One outcome of these changes are an increasingly greater likelihood for adolescent boys to be enrolled in school rather than herding livestock on behalf of the family in a landscape shared with numerous native vegetation and wildlife species.

**Methods:**

This study compared identification and knowledge of native plant species between boys enrolled in school with boys of similar age but primary responsibility as herders, called moran. Study participants walked an approximately 100 m path with 10 flagged points in which they were asked to identify any plant species at that point and associated facts of each species, within a 1 m radius.

**Results:**

On average, moran identified 38 species compared to 20 for students, including nearly 13 (of a possible 15) species considered to have high cultural significance. Students identified an average of 8.6 culturally-significant plants. Further, moran shared nearly 18 correct facts about the plants, compared with ten for students. In addition, herding frequency was the only significant predictor of plant identification in a linear regression.

**Conclusion:**

The results demonstrate that while formal education undoubtedly provides benefits to students, attendance in school in lieu of the traditional role of herders has consequences on young men in Samburu related to ability to identify native and culturally-significant plants. This further shows the importance for communities like those in Samburu undergoing change need to develop alternative options to transmit local traditional knowledge to its younger generations.

## Background

There is widespread recognition in the field of natural resource management that traditional ecological knowledge (TEK) of indigenous communities can positively influence sustainable land use practices [1–4]. In addition, TEK can broaden the way environmental problems are conceptualized and solved, and can add diversity to land managers’ understandings, ultimately bolstering a socio-ecological system’s resilience [5].

In sub-Saharan Africa, the loss of TEK among indigenous communities has been described as one of the greatest challenges to the continent for retaining cultural continuity and achieving sustainable resource management [6]. The loss and lack of recognition of TEK were also cited as two of six of the greatest threats to biodiversity conservation in Africa by the United Nations Development Program Convention on Biological Diversity in 2006. The importance of these issues is paramount, as numerous studies highlight the valuable contributions of TEK in the provision of sustainable East African pastoral socio-ecological systems [7–9]. Several studies also call attention to the problem of TEK erosion [10, 11], but there is need for more research to understand this trend [9, 12–15].

At the broadest of levels, widespread changes in pastoralism cultures and systems are known to be occurring around the globe, driven by climate change, population growth, economic development, land tenure changes and other factors. Dong et al.’s study of 10 pastoral case studies around the world confirmed such widespread change and the increased vulnerability of pastoral systems across the globe as a result, from the Sahel in Africa to the high plateaus of central China to the Andes of South America [16]. Additional drivers of change in pastoral systems include migration, as Rasmussen et al.’s model revealed in their study of the Sahel [17].

Many of these changes and vulnerabilities are true in the Samburu region of northern Kenya as well. As a result, in Samburu, Kenya there is increasing concern that TEK is eroding as successive generations seek less traditional ways of life which tend to favor more modernized ways of living and western knowledge systems [note: Samburu (un-italicized) refers to the place (i.e., Samburu County) whereas *Samburu* (italicized) will refer to the tribe/cultural group. Other tribes/cultural groups will be italicized accordingly]. Specifically, there is concern that as more youth in Samburu pursue formal education, there will be a shift away from pastoralism, and less interest and competence in traditional knowledge as a result. More specifically, in traditional *Samburu* culture, boys typically carry primary responsibility for tending to family livestock. Today, with greater emphasis and value placed on education, these boys are increasingly more likely to be enrolled in school, and many may therefore lack the direct experiences in the landscape as herders that can help facilitate TEK transmission.

The purpose of this research is to assess if attending school in lieu of practicing pastoralism for boys contributes to a difference in knowledge about the traditional names and uses of local plants. We hypothesize that adolescent males who engage in traditional herding activities in lieu of school will identify more local plant species and be more traditional knowledgeable about local plant uses than their school-going peers.

### Review of literature

#### Traditional ecological knowledge

TEK has been the focus of many researchers and therefore comes with many definitions. Raymond et al. defined TEK as “a subset of indigenous knowledge that includes knowledge and beliefs handed down through generations by cultural transmission and which is related to human-environment interactions” (p. 1768, [18]). Fernandez-Gimenez et al. describe TEK as “a system of experiential knowledge gained by continual observation, and transmitted among members of a community,” (p. 306, [19]). In this study, we used a definition from Berkes, Colding and Folke [3]: “a cumulative body of knowledge, practice, and belief, evolving by adaptive processes and handed down through generations by cultural transmission, about the relationship of living beings (including humans) with one another and with their environment” (p. 1252). We selected this definition because it includes an adaptive focus and the rationale for our study in its specific location in Kenya was due to considerable social and environmental change, which will require its people to adapt accordingly.

#### Traditional ecological knowledge and sustainable resource management

TEK is an important component of a number of concepts within community-based natural resource management (CBNRM) and related concepts, including resilience, community participation and stakeholder collaboration [3, 20, 21]. The push over the past 2 decades for natural resource management that honors and integrates indigenous knowledge and traditions is seen as an important step towards sustainable socio-ecological systems [2, 3, 9, 22].

Sustainable land management often requires sufficient collection, retention and transmission of knowledge gained through years of interacting with a landscape [23]. For example, in her study on *Maasai* vegetative knowledge (a tribe closely related to the *Samburu*), Kiptot found that the tribe possessed complex knowledge about grazing which has supported sustainable landscape management over many generations [9]. Similar knowledge systems have evolved within nomadic pastoral communities throughout the world, where herders base decisions on intimate and culturally-embedded knowledge of their environments passed on between generations [8, 19, 24]. If intergenerational transmission of this knowledge is weakened, resilience may be lost and successive generations may be less equipped to contend with new environmental challenges as they arise [5].

#### Transmission of TEK

TEK transmission is the transfer of traditional knowledge between individuals of a particular indigenous group. The primary modes of transmission are dynamic, varying with place and across time, though it commonly occurs through direct interaction with one’s environment [12, 25]. Many individuals within a culture learn through direct experiences as exemplified in a study of Luo children in western Kenya who learned ethno-medicinal practice through the process of contending with their own illnesses, with guidance from peers and adults [13]. TEK is also often conferred during normal social interaction, and by oral transmission through story-telling [3, 12].

While TEK transmission most often occurs between older and younger generations, or *vertical* instruction, it can also occur through more *horizontal* interactions between peers, and *oblique* transmission from non-familial mentors [26–29].

Communities have shifted to alternative modes of TEK transmission when traditional vertical and horizontal avenues are weakened, or as more viable alternatives develop. These new and non-familial avenues for TEK transmission have often emerged when communities undergo social and economic changes. In their study on ethnobotanical knowledge transmission in a rural Patagonian community, for example, while knowledge specialists (*shamans*) played a significant role in TEK transmission in the past, familial transmission had emerged as a more contemporary option as shamans became a less common role in the community [30]. Similarly, Tyrolean (Austrian) chefs have become a source for traditional food knowledge transmission to employees in restaurants who would have acquired similar knowledge in the past from family members [27]. As some Tanzanian communities change to market-based economies, community produce markets can facilitate knowledge transmission about local vegetation and foods, where merchants exchange plant knowledge with one another through the course of selling their respective goods [14].

#### Factors affecting TEK transmission

Loss of TEK has been attributed in part to Western influences including prevailing models of formal education, medicine, political systems, religion and technology [22, 27, 31, 32]. These factors were corroborated by a report from the United Nations Environment Programme (UNEP) in 2006 which presented a list of 23 barriers to traditional knowledge transmission in Africa, including loss of or dramatic change to ecosystems, poverty, climate change, immigration and emigration, schools, urbanization, and others [12]. Such factors can contribute to TEK loss through a number of ways: by catalyzing a shift in cultural norms, increasing the availability of western medicine, emphasizing western knowledge over other ways of knowing, reducing the amount of time young people spend in the natural environment, and biodiversity loss from development or land conversion [14, 32, 33]. For example the erosion of ethno-medicinal knowledge among young *Tsimane*’ men in Bolivia was attributed to the partial adoption of western medicine and stigmatization of traditional diets [32]. For reference, Zent provides an extensive overview of research pertaining to these sources of TEK erosion [34].

These factors can influence TEK transmission differently between communities, so the extent of these effects must be studied on a community-by-community basis in order to determine the best strategies for mitigating TEK loss and/or devaluation in a specific area [28]. For example, while formal Westernized education systems can be a contributor to TEK erosion, its value to facilitate TEK transmission can also be positive, depending on the community [28].

#### Formal education & TEK

While formal education can have a negative impact on TEK, research has shown that the nature of this relationship varies considerably, including results that support the argument that western approaches to education can actually help pastoralism successfully adapt to widespread change [35]. Overall, the research on TEK and formal education remains mixed. For example, a study among Taiwanese youths found that TEK knowledge was negatively correlated with the number of years a person had been in school [36]. Another study in Columbia and Guatemala concluded that formal education was a major factor contributing to the degradation of intergenerational TEK transmission [12]. Others have found similar evidence of this negative relationship between TEK and formal education [33, 36, 37]. Explanations for the negative effect of formal education on TEK include the constraints that formal education places on a student’s time and a dismissiveness of traditional knowledge [25, 28].

Conversely, in other research the relationship between formal education and TEK erosion is more ambiguous. Among rural communities in Tanzania, a weak correlation between the number of years a person spent in school and their knowledge of medicinal plants was found [14]. Among the Piaroa people of Venezuela, on the other hand, researchers found a strong positive correlation between age and plant knowledge (explained by the natural acquisition of knowledge over time), and only a weak negative correlation with the number of years a person spent in school, concluding that formal education does seem to explain a certain degree of TEK erosion [38]. Similarly incongruent findings are reported elsewhere [28, 39].

Some studies have shown that education is not a strong explanatory variable for TEK erosion at all [40, 41]. By comparing plant knowledge of children of different generations (i.e., 40 years apart), research showed that plant knowledge has persisted in spite of several decades of modernization and increased formal education, and that formal education can even help in supporting pastoralism systems [42]. A mixed methods study in Western Nigeria found minimal support for a negative correlation between formal education and TEK knowledge, and the authors’ qualitative work noted that formal education was essential for retaining some aspects of TEK [26]. Further, a study in the high Andes found that a participant’s level of formal education had no effect on plant knowledge, and concluded that individual motivation was a more influential factor in retention or loss of TEK [41]. Given the place-based circumstances that influence TEK retention and loss, it is incumbent to examine TEK issues on a community-by-community basis [25].

#### Study site

**Fig. 1:**
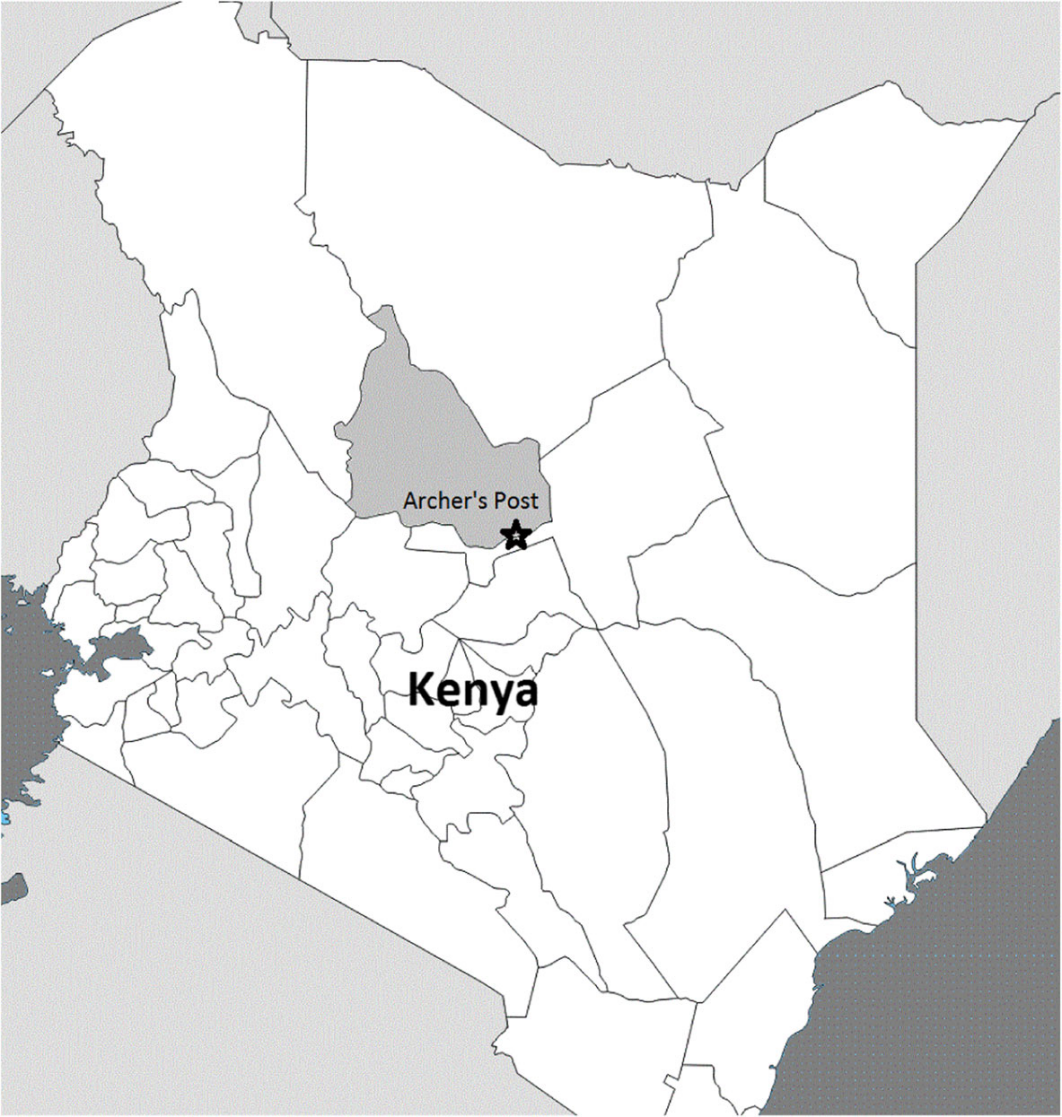
Archer’s Post, Samburu County, Kenya

This study focused on communities within Archer’s Post in Samburu County, situated along the Ewaso Nyiro River in northern Kenya (see Fig. 1). Archer’s Post is the largest settlement within the federally-designated Waso Ward [formerly of the Samburu East District, population 59,000. It is characterized by the relatively flat expanses of *Acacia-Commiphora* bushland north of the Ewaso Nyiro River and south-east of the nearby highlands.

Archer’s Post is a market town that serves as a center of commerce for its permanent residents as well as the nomadic and semi-nomadic pastoralists of the surrounding areas. The arid acacia bush-land and lack of infrastructure and amenities surrounding the town historically limited the primary economic activity to nomadic pastoralism. Increasingly, in the wake of ongoing development and improvements, the town now provides alternative livelihood opportunities, mostly in the forms of trade, day labor, and services for tourists.

Many families have moved to Archer’s Post specifically to send children to school, according to local informants. It has the largest concentration of primary schools in the area, and the only secondary school in the Waso Ward. Kenya’s school system is modeled after that in the United Kingdom. Students attend (free) primary school for 8 years (standards 1–8), and can continue to 4 years of secondary school and 4 years of college at their own expense. Admittance into secondary school (and college) is contingent upon national standardized exam scores, so much of the instruction is designed around a nationalized curriculum and exam.

The population of Samburu consists largely of *Samburu* and *Turkana* ethnic groups, with some migrant *Somali* and *Borana* families. Traditionally, *Samburu* males in their early-to-mid-teenage years assume the roles of moran. At this stage in life, the moran function primarily as warrior guardians of their community and its livestock. As traditional nomadic pastoralists, they frequently embark on long cattle herding journeys that can last for months. During this time they gain knowledge about the natural environment, including its vegetation, topography, and wildlife as well as the ecological effects of their interaction with it. This is known to contribute to the adaptability and resiliency of these and similar pastoral populations [7, 24].

Ethnobotanical knowledge remains important to daily life both in, and outside of the context of pastoralism in Samburu. Even in permanent settlements in Samburu, traditional remedies are still used for cases such as skin infections and post-natal care [43, 44]. Local plant resources also remain commonly used in construction, tool-making, nutritional supplementation and for cultural adornment [43, 44].

Comprehensive research of the ethnobotanical importance of plants for Samburu revealed findings that are relevant to the context of our study [43]. This study identified 449 plant species, and in interviews with *Samburu* in the Mount Nyiru region, noted that 249 of the species had specific utility value to *Samburu*, mostly as fodder for livestock or for medicine. However, the study also noted that changes leading to a less nomadic lifestyle were less in his study site than other regions of Samburu, such as the site for our study.

A second highly relevant study involved 100 individuals in Samburu from a wide range of ages and livelihoods who participated in interviews and plant walks for the researchers to assess and compare traditional plant use and knowledge [44]. Their study revealed that older participants could identify and share more knowledge about local plants than younger participants, and that anthropogenic factors (including transition from nomadic to settled lifestyles) were contributing to declining plant knowledge.

## Methods

### Community-based research

This study was guided by a number of principles of participatory and community-based research. The community’s concerns about TEK erosion were originally expressed in a previous 2006 project [45]. Stakeholders in the current study were consulted at the inception (e.g., identification of the problem and research question) and throughout the research, following advice from the continuous engagement model [46]. Local Samburu community members participated as researcher assistants and were empowered to influence data collection design and analysis based on cultural appropriateness and local need.

Fieldwork began by identifying plants, in partnership with local elders (i.e., *wazee*), that were considered most important to the *Samburu* and *Turkana* people in the Waso Ward. This was conducted via small group meetings. Many of these species were also identified as locally significant for their ethnobotanical value based on prior research and interviews with local wazee [42, 44]. These plants were located, photographed and identified by their scientific names with the help of local plant experts and a botanical expert from the University of Nairobi. This information was then used to select a site for a 30 m plant trail which served as part of our research strategy (see below). In addition, several meetings were held with wazee to elicit their thoughts about TEK and barriers to its transmission.

### Sample

This study compared young men who identified as traditional moran with peers enrolled in school. Traditional moran spend significant time in the natural environment tending to livestock, often covering long distances in a landscape shared with an abundant population of wildlife species.

We aimed to sample 30 students from each of the two groups, based on available resources. Male students were sampled from standards 7 and 8 from the three largest public primary schools in Archer’s Post. The students were assigned a number, and 33 were randomly selected. Moran were not able to be randomly selected, due to their nomadic and semi-nomadic nature, and instead were selected via convenience sampling from the livestock market area of Archer’s Post, based on their willingness to participate. All moran reported being from within 25 km of Archers Post. A total of 33 were selected, and 26 completed their participation in the study.

### Data collection

Plant identification and associated plant facts were used as proxies to measure TEK from the two groups, a practice common within TEK research [12, 22, 28, 36]. A site was identified along a vegetated riparian area of the Ewaso Nyiro River and 10 points along a designated 100 m path were flagged, in which all 15 of the plant species determined to be of cultural value by wazee were present. Participants were accompanied by at least one researcher or research assistant who asked them to stand at each point and name all of the plants they could identify within a one meter radius. When participants identified one of the 15 target plants, they were asked to provide any facts about its local uses. The time to complete the activity varied between 30 and 120 min for each participant. This approach is similar to the methods employed in other studies [37, 42] In addition, participants provided basic demographic information such as their age, tribal affiliation, primary area of residence, years spent in school, and frequency of herding on a scale of one (1; never) to five (5; daily).

### Data analysis

Data was analyzed along three major parameters: number of total species correctly identified, number of important species (out of 15, as noted by wazee) identified, and number of total correct facts the students reported about the 15 important species. Facts were determined as correct in partnership with the wazee, who shared abundance of information about each of the 15 species and which was used to rate responses. Facts included information about how plants were used locally such as for medicine, grazing, household use (e.g., charcoal, construction), food and similar uses.

Responses were entered into a statistical software package (i.e., SPSS v. 21), and descriptive statistics and comparisons between students and moran (via independent sample t-tests) were conducted. In addition to student/moran group comparisons, we examined which independent variables were most likely to predict plant identification and knowledge, via a multiple regression.

## Results

### Plant identification

A total of 33 students and 26 moran participated. Moran identified more overall plant species than students with mean counts of 38 and 20 plants, respectively (*p* < .01; see Table 1).

**Table 1:** Average number of total plants identified for students and moran

		Students/Moran		*t*-value	*p*-value
		Students	Moran		
Plants identified	x̅	20.18	38.04	7.15	<.01
	sd	9.78	9.19		

Of the 15 specific species identified by wazee as being culturally important, *moran* were able to identify an average of 13 and students identified an average of nine (*p* < .01; see Table 2).

**Table 2:** Difference between number of culturally significant plants identified by students and moran

		Group		*t*-value	*p*-value
		Student	Moran		
Total # culturally important species identified^a^	x̅	8.61	12.87	6.30	<.01
	Sd	3.32	1.69		

^a^Out of a maximum of 15

Significant differences in the ability of moran and students to specifically identify each of the 15 culturally important species were found among 11 of the 15 species. No differences were found with identifying Ltepes (*Acacia tortilis*; *p* < .38), Lordanyai (*Loranthus sp*., *p* < .08), Lparuai (*Hyphaene thebaica, p* < .07) and Sukuroi (*Aloe secundiflora*; *p* < .07). For other species, differences in ability to identify was significant. Moran were more likely to identify nine of the 15 species (*p* < .01). None of the plant species were identified more often than students (see Table 3).

**Table 3:** Differences in percentage of students and moran that correctly identified *culturally important* plants by species

Species: Samburu (Turkana) name *Scientific name*	Correctly identified^a^		*Chi-square*	Sig.
	Student	Moran		
Lawai (Ekurchanalt) *Delonix elata*	81.3 %	100 %	5.04	<.03
Ldupai (Emojo) *Sanserveria intermedia*	68.8 %	100 %	9.13	<.01
Lgiriai (Ethajait) *Lawsonia inermis*	75 %	100 %	6.73	<.01
Lpupoi (Engumo) *Grewia villosa*	81.3 %	100 %	5.04	<.03
Lominira (Ekaretrete) *unknown*	18.8 %	95.2 %	29.68	<.01
Lordanyai (Lorkirdanyai) *Loranthus sp.*	21.9 %	43.5 %	2.92	>.08
Lparuai (Engwael) *Hyphaene thebaica*	87.5 %	100 %	3.23	>.07
Ltepes (Etiir & Ewoi) *Acacia tortilis*	96.9 %	100 %	0.76	>.38
Ngirman (Egong) *Hildebrandtia sepalosa*	32.3 %	69.6 %	7.88	<.01
Sakurdumi (Emoni) *Kedrostis gijef*	18.8 %	62.5 %	11.20	<.01
Sakurtuti (Lobara) *Cissus quadrangularis*	9.3 %	58.3 %	15.55	<.01
Salapani (Etuntun) *Cordia sinensis*	71.9 %	100 %	7.73	<.01
Serichoi (Edung) *Boscia coriacea*	53.1 %	95.5 %	11.20	<.01
Sukuroi (Echuchuka) *Aloe secundiflora*	87.5 %	100 %	3.10	>.07
Sumanderi (Ethimanderi) *Commiphora schimperi*	59.4 %	95.8 %	9.72	<.01

^a^Percentage of participants in social group (student of w) that correctly identified species

### Plant knowledge

When a participant identified one of the 15 culturally-significant plant species, they were asked to share any information about the species. Moran recalled a statistically significant higher average number of overall facts (x̅= 17.87) than students (x̅= 10.28; *p* < .01; see Table 4).

**Table 4:** Average number of facts recalled about culturally important plants by students and moran

		Group		*t*-value	*p*-value
		Student	Moran		
Total # facts^a^	x̅	10.28	17.87	3.63	<.01
	Sd	6.18	9.32		

^a^Total number of correctly recalled facts about plants across all 15 species

### Regression analysis

A backward regression analysis resulted in one significant independent variable, herding frequency, that explained 30 % of the variability in total plant identification (adjusted R-squared; *p* < .01; see Table 5).

**Table 5:** Assessment of herding frequency as a predictor of plants identification

	Standardized regression coefficient (β)	*t*	Sig.	*F*	Adjusted *R* ^*2*^
Constant		1.28	.21	22.42	.30
Herding Frequency	.56	4.74	<.01		

## Discussion

The results of this study are consistent with previous research: herding can help facilitate TEK transmission. Regression results indicated that herding frequency predicted an individual’s ability to identify plant species more than any other factor. This is corroborated by many results showing that moran could identify more of the plants in this study, and knew more about plants they identified.

In informal follow-up conversations with moran and wazee following analysis to elicit explanations for the results, moran described herding with peers (i.e., horizontal transmission) and elders (i.e., vertical transmission) when asked to think about how they gained this knowledge. In many instances during these discussions, moran described practical, experiential herding moments situated in the landscape when a plant’s name and/or knowledge was shared by a peer (i.e., other moran) or mentor (i.e., father). The combination of the study results and these informal discussions supports an assumption that herding provides a common mechanisms by which young males gain ethnobotanical knowledge in this region.

If herding serves as a conduit for TEK acquisition and students herd much less frequently than moran, then it is reasonable to conclude that school attendance, compared to herding, may inhibit TEK transmission unless a viable substitute (to herding) is identified. This notion is further supported by the results that moran identified more than twice as many plants as students, and on average shared 70 % more facts about traditional uses of key plant species.

Our findings also suggest that TEK erosion is likely occurring among boys who regularly attend school in our study (in lieu of herding). In this community, education is increasingly desired by families for their children; enrollment in the three long-existing local primary schools was among its highest-ever levels according to school leaders. Two additional primary schools were recently added in the community, and numerous key informants spoke of the changing value on education within the community during the past generation.

During our conversations, wazee expressed concern that students lacking TEK was indicative of a broader loss in appreciation for local customs and culture more broadly. This led to recognition that they may have a new role in their community, as active educators and advocates about local culture, including ethnobotanical knowledge, traditional grazing practices and similar concepts. Taking on such new roles in the community to encompass TEK transmission through non-traditional means (e.g., activities with school environment clubs) shows a willingness by wazee to be adaptive, and how such willingness has perhaps led to the resilience that has persisted in this culture and landscape for generations.

The wazees’ concern with TEK erosion also signifies a potential shift in community power dynamics. Elders are the current bearers of traditional knowledge, but whether such knowledge will be valued by a growing non-herding class of males is uncertain. This could be alleviated if the importance of TEK is conveyed to school-going young people. Moreover, TEK and herding have long been at the core of what it means to be *Samburu* or *Turkana*, so maintaining TEK is potentially a matter of retaining an important aspect of cultural pride.

Based on our observations, schools in this region and throughout Kenya almost exclusively teach based on western knowledge systems, and the teaching profession in Kenya generally does not promote approaches such as place-based education (where TEK might be integrated) [47]. Additionally, teaching positions are governed by the national Ministry of Education, meaning schools in a place such as Samburu can be assigned teachers from vastly different parts of the country and with minimal knowledge about the local environment.

Given that students enrolled in school possess less ethnobotanical knowledge than their herding peers, and that such knowledge is regarded by the wazee as essential to *Samburu* and *Turkana* cultures, there is a need to develop alternative modes of transmission for students. Most of the educators we spoke with, including administrators and district education personnel, were not aware that their students had low levels of TEK compared to moran, or that TEK loss by students was a concern of elders in their community. Although formal education has been implicated as a barrier to TEK transmission in other indigenous communities it has also been suggested in the literature that western and traditional pedagogies are not inevitable competitors [25, 28, 47, 48]. In fact, researchers contend that with leadership and willingness to adapt, formal education can be leveraged to facilitate TEK transmission [47, 48].

One argument for a simple strategy to ensure that younger generations are exposed to traditional knowledge would be to bring that knowledge into formal education. By providing opportunities in the curriculum or during school-sanctioned extracurricular lessons, TEK experts (e.g., moran and wazee) and educators can help integrate TEK as a traditional way of knowing, and present ethnobotanical knowledge along with more conventional scientific knowledge in schools.

In conjunction with this study, the research team and local collaborators convened wazee, educators and conservation professionals to develop pilot programs that targeted Samburu students. Among the projects was a plant identification guide featuring *Samburu* and *Turkana* ethnobotanical knowledge that was distributed in schools. Selected students were taken on a three day field trip into nearby protected areas where they were given hands-on, place-based TEK instruction from local wazee and supported by educators and conservation staff from non-governmental organizations. The participating students later assisted the elders in a series of after-school programs with their peers, where they brought plant samples to the classroom, and co-delivered ethnobotanical lessons with wazee. The success of these efforts were not measured as part of this study, but participating elders expressed satisfaction that they were provided an opportunity to convey information about local plants to non-herding youth in their community.

As described earlier, the effect of formal education on TEK is mixed with some studies indicating education is a barrier to TEK while other studies showed no adverse effect. This study supports the notion that formal education diverts students from herding where they traditionally acquire ethnobotanical knowledge. This was an unintended outcome of greater access to and value on education, and points to the need to look at TEK through a system lens.

## Conclusion

As an integral part of the community’s semi-nomadic traditions, and a marker of cultural pride, TEK is an important part of maintaining cultural cohesiveness and sustainable natural resource management in Samburu. The erosion of TEK among students is suggestive of a pervasive social rift that may impact community dynamics and negatively influence people’s valuation of, and ability to implement Samburu’s traditional, resource utilization practices. It also stands to reason that the sustainable management of local plants, and ultimately plant biodiversity in the area, may suffer if medicinal plant species are no longer perceived to be of significant value by the broader community [6].

One potential strategy for addressing the decline in TEK among students in Samburu (and perhaps other regions of Kenya) may be to leverage the formal education system to advance the legitimacy of TEK (and traditional management strategies). Formal education is, in essence, systemized knowledge transfer, so it seems like an obvious and readily available channel for renewed TEK transmission. Schools in Waso East could continue to collaborate with local TEK experts and elders to incorporate TEK lessons into extracurricular activities. There are also opportunities to work with the various conservation entities in the area to develop TEK related place-based education programs [25, 46].

### Limitations

Moran were recruited for this study from one relatively small community situated in a geographically large area. This created an opportunity for sampling bias, as moran in a given area are typically a tightly-woven social group, and information is often shared within that group. Moran from other areas may have a different level of knowledge or different information about plants.

### Suggestions for future research

This was not a longitudinal study so it remains uncertain whether knowledge has actually been lost *over time*. A follow up study, using the data from this study as a baseline could help reveal if such trends exist, especially regarding TEK erosion among the moran.

This study was designed to look at herding, a predominantly male activity, as an important factor that is limited by the formal education system. Similar research could also assess whether similar trends exist among females in Samburu.

Additional analysis of our data hints at the possibility that students may acquire enough TEK after completing primary school to catch up with their moran peers; an idea that also merits further research.

## Abbreviations

TEK: Traditional ecological knowledge
UNEP: United Nations Environment Programme
